# Reduced TRPM8 expression underpins reduced migraine risk and attenuated cold pain sensation in humans

**DOI:** 10.1038/s41598-019-56295-0

**Published:** 2019-12-23

**Authors:** Narender R. Gavva, Robert Sandrock, Gregory E. Arnold, Michael Davis, Edwin Lamas, Chris Lindvay, Chi-Ming Li, Brian Smith, Miroslav Backonja, Kristin Gabriel, Gabriel Vargas

**Affiliations:** 1 Amgen Inc., Thousand Oaks, California, USA; 2 Worldwide Clinical Trials, Morrisville, North Carolina, USA

**Keywords:** Ion channels in the nervous system, Sensory processing

## Abstract

Multiple genome-wide association studies have identified non-coding single-nucleotide variants (SNVs) near (e.g., rs10166942[C]) or within (rs17862920[T]) the TRPM8 gene that encodes a cold thermosensor is associated with reduced migraine risk. Furthermore, rs10166942[C]) and rs10166942[T]) are more prevalent in populations that reside in hotter and colder climates, respectively. Here we assessed whether these alleles affect *TRPM8* expression in humans and human physiologic responses to cold challenge. Here we show that TRPM8 expression is decreased from the chromosome harboring the rs10166942[C] allele in the human dorsal root ganglia. Moreover, carriers of rs10166942[C] required significantly lower temperatures and longer duration of exposure to reach a cold pain threshold (CPTh), which correlated with decreased TRPM8 expression expected in the carriers. This study provides evidence for a genotype-dependent influence on cold pain sensation suggesting that carriers of the reduced migraine risk allele have reduced sensitivity to cold stimuli and that TRPM8 acts as a cold thermosensor and cold pain transducer in humans. Reduced TRPM8 expression and function underpins the migraine protection in carriers of rs10166942[C]; thus, the evaluation of TRPM8 antagonists as migraine therapeutics is warranted. Furthermore, these results provide mechanistic insights for evolutionary positive selection of rs10166942[T] allele in adaptation along latitudinal cline to colder climates.

## Introduction

Transient receptor potential melastatin 8 (TRPM8) is a non-selective cation channel that is activated by cool temperatures and compounds that elicit cool sensation, such as menthol and icilin (AG-3-5), and that serves as a cold thermosensor *in vivo*^1–4^. Knockout mice studies indicated that TRPM8 is required for sensing innocuous ambient cold temperatures^1,2,5^. Role of thermosensory channels in thermoregulation have been demonstrated initially with agonists: TRPV1 activation caused a drop whereas TRPM8 activation caused an increase in body temperature^6,7^. Further, antagonists demonstrated a basal tone of activation because antagonists by themselves altered body temperature: TRPV1 antagonists increased body temperature in rodents, dogs, non-human primates and humans whereas TRPM8 antagonists decreased body temperature^8–10^.

TRPM8 is highly expressed in migraine and pain neuronal circuitry, such as trigeminal and dorsal root ganglia (DRG)^3,4^. Multiple genome-wide association studies have identified non-coding single-nucleotide polymorphisms (SNP) in cis or within the TRPM8 gene (rs10166942[C] is located 950 bp 5′ to the start codon and rs17862920[T] is located within the first intron) are associated with reduced migraine risk (odds ratios of 0.78 and 0.77, respectively)^11–13^. In this study, we have assessed the extent to which reduced migraine risk alleles affect *TRPM8* expression by evaluating allelic expression imbalance (AEI) at the transcript level using next generation sequencing. We have also investigated if reduced TRPM8 expression is associated with alterations in physiologic responses to cold challenge.

## Results

**Figure 1:**
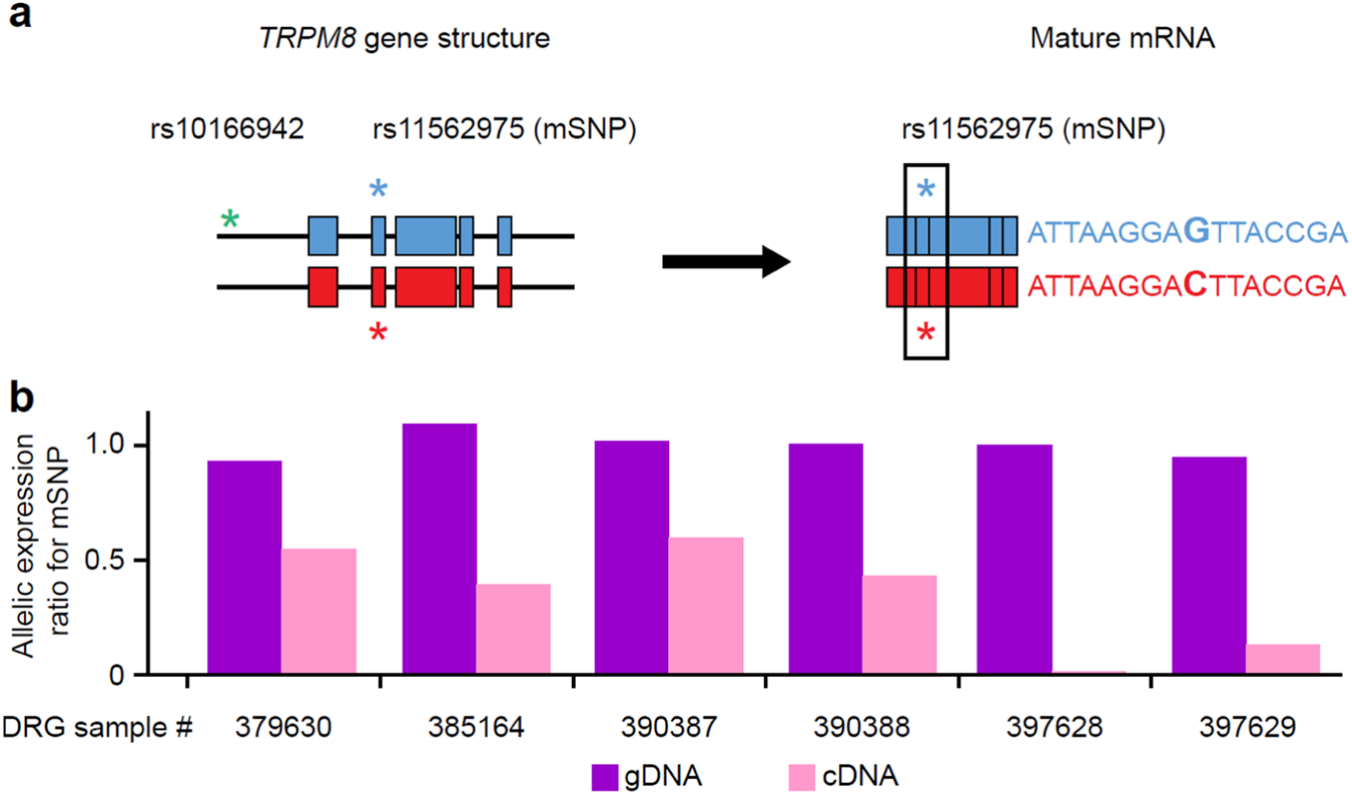
Detection of *TRPM8* allelic expression imbalance using next generation sequencing. Schematic diagram of *TRPM8* gene structure on parental chromosomes and subsequent allelic mRNA transcripts (**a**), and allelic expression imbalance of *TRPM8* mRNA from DRG samples harboring the reduced migraine risk alleles rs10166942[C] and rs17862920[T]. (**b**) Minimal allelic imbalance was observed at the genomic level (magenta bar; ratio minor allele counts divided by common allele. A ratio of 1 = no imbalance). Significant imbalance was observed at the allelic transcript level in all DRG samples (light pink bar). A ratio of 0.5 denotes a 50% reduction in minor allele expression. Both rs10166942[C] and rs17862920[T] are present on the same chromosome in DRG sample 397628 while other samples have only rs10166942[C].

We assessed if reduced migraine risk alleles would influence *TRPM8* expression by evaluating allelic expression imbalance (AEI) at the transcript level using next generation sequencing. To measure allele-specific mRNA expression, we identified three marker SNPs (mSNP; rs11562975[C], rs28901637[T], and rs13004520[C]) showing sufficient heterozygosity within a coding region of *TRMP8* (het-mSNP) (Fig. 1a and Supplementary Table 1). DRGs from 15 human donors were genotyped to identify donors that were heterozygous for one of the mSNPs as well as one of the reduced migraine risk SNPs (het- rmrSNP, rs10166942[T/C] and rs17862920[C/T]). Six donors’ DRGs were heterozygous for both rs10166942 alleles (T/C) and one of the three mSNPs and were subsequently used to determine allele-specific mRNA expression (Supplementary Table 2). One DRG showed heterozygosity for both rs10166942(T/C) and rs17862920(C/T). Small regions of genomic DNA (gDNA) on which the het-mSNPs and het-rmrSNPs reside were amplified by polymerase chain reaction (PCR) and assessed for an imbalance at the genome level to control for false signals caused by aneuploidy or amplification bias. We detected minimal imbalance for all heterozygous SNPs at the gDNA level (Fig. 1b and Table 1).

**Table 1:** mSNP imbalance in the *TRPM8* gDNA region and *TRPM8* cDNA in DRG samples heterozygous for reduced migraine risk SNPs rs10166942 and rs17862920.

DRG sample ID	SNP ID	Average reads (gDNA)	Average allelic imbalance (gDNA)^a^	Average reads (cDNA)	Average allelic expression imbalance (cDNA)^a^	Absolute fold change in allelic expression^b^
379630	rs11562975	19,899	0.98	22,034	0.57	1.7
385164	rs11562975	16,624	1.14	30,039	0.41	2.4
390387	rs11562975	21,689	1.06	19,849	0.63	1.6
390388	rs28901637	735,899	0.95	632,781	0.41	2.4
397628	rs13004520	460,612	1.02	148,654	0.01	99.0
397629	rs11562975	409,588	0.94	367,027	0.12	8.2

^a^Number of reads for minor allele divided by number of reads for common allele at the gDNA or transcript levels. ^b^Fold change in allelic expression regardless of which allele is increased in expression. cDNA, complementary DNA; DRG, dorsal root ganglia; gDNA, genomic DNA; SNP, single-nucleotide polymorphism.

To evaluate AEI at the transcript level, amplicons containing the het-mSNPs were generated from complementary DNA. Strikingly, imbalanced expression of the allelic het- mSNPs was observed in all six DRGs, with differences in allelic expression ratios ranging from 0.01 to 0.63, always reduced TRPM8 expression from the chromosome harboring rs10166942[C] (where a ratio of 1 indicates there was no imbalance; Fig. 1b and Table 1). Remarkably, the sample harboring both het-rmrSNPs showed the highest level of AEI, i.e., dramatic decrease in TRPM8 expression from the chromosome harboring both rs10166942[C] and rs17862920[T]. We confirmed that the rmrSNP allele rs10166942[C] and the mSNP allele with lower expression were co-located on the same chromosome using haplotype phasing (long-range PCR and Sanger sequencing). In five DRGs, the rmrSNP allele (rs10166942[C]) was linked to one of the mSNP alleles (either rs11562975[C], or rs28901637[T], or rs13004520[C]) that showed decreased *TRPM8* expression (Supplementary Table 3). One DRG sample was not amenable to long-range PCR because of gDNA degradation. These data indicate that the reduced migraine risk allele *rs10166942[C]* is associated with reduced *TRPM8* expression.

Because TRPM8 is a cold thermosensor, we hypothesized that carriers of rs10166942[C] may be less responsive to cold challenge. It has been shown that pharmacologic blockade of TRPM8 attenuates autonomic and behavioral cold defense mechanisms^8^ and cold pain sensation in the cold pressor test (CPT)^14^. Therefore, alterations in physiologic responses to cold challenge may reflect an association with reduced TRPM8 function.

To investigate this further, we enrolled 38 healthy male volunteers in a Phase 0 study, and evaluated the impact of rmrSNP alleles on cardiovascular reactivity and cold sensitivity based on cold pain threshold [CPTh] and tolerance [CPTo]) using CPT and quantitative sensory testing.

Individuals (mean age of 29.6 years; 92% of European ancestry) were separated into two cohorts: carriers who had rmrSNP alleles (*n* = 18), or non-carriers who were homozygous for the common SNP allele (*n* = 20). Reduced migraine risk genotype distribution and SNP group status (whether they were carriers or non-carriers of the aforementioned SNP) did not have any bearing on their CPT eligibility criteria, suggesting that enrollment was genetically unbiased relative to the test alleles (Supplementary Table 4). In the carrier group, 11 individuals were heterozygous (T/C) and seven were homozygous (C/C) for the rs10166942 reduced risk allele. For rs17862920, 10 individuals were heterozygous (T/C) and one was homozygous for the reduced risk allele (T/T). Individual genotyping results showed that all individuals with reduced migraine risk allele rs17862920[T] were also carriers of rs10166942[C], demonstrating that rs17862920 is in haplotype with rs10166942.

**Figure 2:**
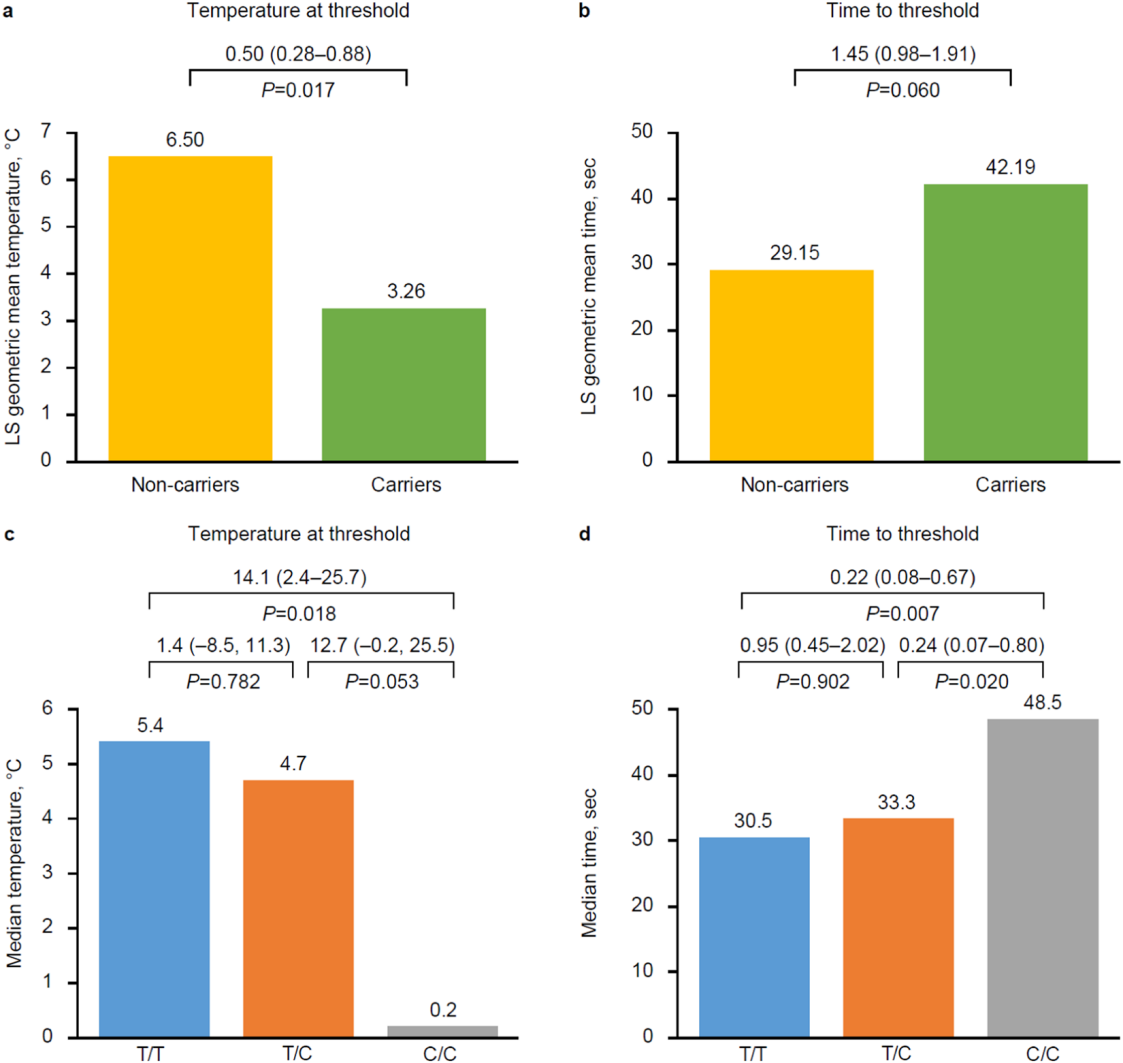
Comparison of QST measurements for rs10166942 between carriers (allele [C]; *n* = 18) and non-carriers (allele [T]; *n* = 20) (**a**,**b**), and between genotypes (**c**,**d**). QST was performed using a Peltier thermode placed against the skin, which was then cooled at a rate of 1 °C per second (until 0 °C). The temperature and time at which the participant experienced the cold pain threshold were recorded. Differences between the groups are represented as means (or medians) with 95% confidence intervals.

No statistically significant differences in systolic or diastolic blood pressure were observed at the group level during the CPT. A potential genetic influence on sensitivity to cold stimuli was observed during QST, wherein carriers had significantly lower CPTh than non-carriers (3.26 °C vs. 6.50 °C, *P* = 0.017; Fig. 2a). Carriers also took more time to reach CPTh than non-carriers after QST challenge, although the difference was not statistically significant (42.2 seconds vs. 29.2 seconds, *P* = 0.060; Fig. 2b). However, no difference was observed in time taken to reach CPTh between carriers and non-carriers during the CPT (Supplementary Table 5). Regarding the analyses for QST CPTo, pain intolerance was generally not reached and nearly all temperature and time observations were recorded as 0 °C and 152 seconds, respectively.

Based on these initial findings, we further evaluated the potential for a genetic influence on physiologic responses to cold challenge by repeating these analyses at the genotype level. The lack of cardiovascular reactivity during the CPT was confirmed at the genotype level (Supplementary Figs. 1 and 2). We found that the reduced cold sensitivity observed in carriers at the group level was driven by individuals who were homozygous for rs10166942[C]. These individuals reached a lower temperature at CPTh compared with individuals homozygous for the common allele (T/T) after QST challenge (0.2 °C vs. 5.4 °C, *P* = 0.018; Fig. 2c). These individuals also reached a lower temperature at threshold compared with heterozygotes (T/C), but a statistically significant difference was not found (*P* = 0.053). Individuals homozygous for the common allele and heterozygotes generally shared a similar response suggesting the allele associated with reduced migraine risk is recessive. Similarly, individuals homozygous for rs10166942[C] took longer to reach CPTh than homozygotes for the common allele after QST challenge (48.5 seconds vs. 30.5 seconds, *P* = 0.007; Fig. 2d). Homozygous carriers of rs10166942[C] also took significantly longer to reach CPTh compared with heterozygotes (T/C) (*P* = 0.020). In the CPT, homozygous carriers of rs10166942[C] had delayed onset of cold pain compared with individuals homozygous for the common allele (T/T), although a statistically significant difference was not detected (*P* = 0.59; Supplementary Table 6).

We found that Likert pain scores were insensitive to genotypic differences in the CPT and QST at the group and genotype levels (Supplementary Tables 6–8). One possible explanation for this observation is that reduction in TRPM8 expression may only attenuate cold temperature sensation but not an overall pain response, because TRPM8 predominantly functions as a cold thermosensor. Full TRPM8 antagonism may be required to observe a reduction in pain as observed with a TRPM8 antagonist^14^. During the study, treatment-emergent adverse events (nausea, dizziness and flushing) were reported in one participant (2.6%) in the non-carrier group.

Although preclinical studies have shown that TRPM8 antagonists prevent CPT- induced blood pressure increases^15^, carriers of reduced-migraine risk alleles did not show any difference in the present study. The lack of change in CPT-induced blood pressure increase between carriers and non-carriers may be explained by observation that cold temperature sensation was attenuated rather than completely impaired.

## Discussion

Previous studies have shown that genetic variation in cis (rs10166942[C]) or within (rs17862920[T]) the *TRPM8* gene are associated with reduced risk of migraine^11,12,16^. Based on AEI analysis, we present haplotype data suggesting that decreased expression of *TRPM8* is associated with a reduced risk for migraine. Our study provides evidence for a genotype-dependent influence on cold sensation suggesting that carriers of the migraine- protective allele have reduced sensitivity to cold stimuli. Homozygous carriers of rs10166942[C] were less sensitive to cold pain suggesting that TRPM8 indeed acts as a cold thermosensor and involved in cold pain sensation in humans.

TRPM8 expression is reduced from the chromosome harboring rs10166942[C] in all the carriers in the range of 47% to 99% with the maximum decrease observed from the chromosome that harbors both reduced risk migraine alleles (rs10166942[C] and rs17862920[T]). The factors that underpin the variability in the magnitude of reduction in TRPM8 expression in carriers of rs10166942[C] are unknown. Since most of the effect on cold pain sensation driven by the homozygotes for the rs10166942[C], we expect a large effect size for migraine protection with full antagonism of TRPM8 supporting an evaluation of TRPM8 antagonists as migraine therapeutics.

**Figure 3:**
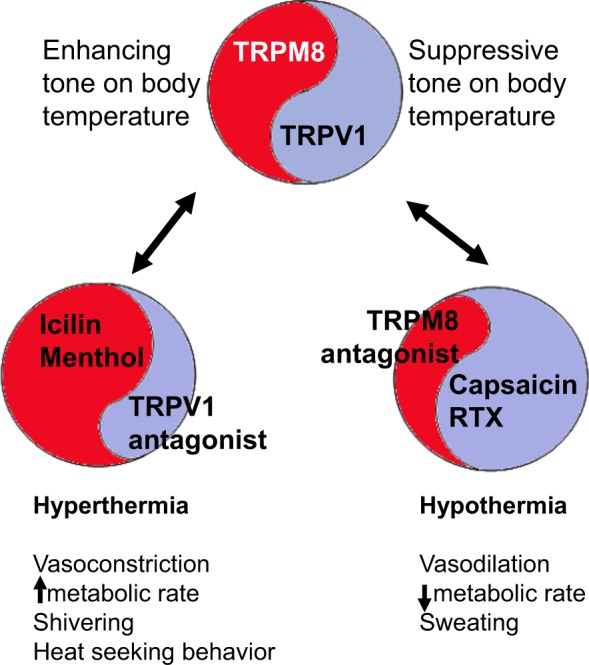
TRPM8 and TRPV1 are part of multiple thermoregulators that balance body temperature in a homeostatic manner. Multiple tonically active ion channels that are further activatable by discrete temperature ranges and chemical ligands are hypothesized to regulate thermoeffectors for homeostatic thermoregulation. TRPV1 has a suppressive tone on the body temperature and TRPM8 balances such an effect by an enhancing tone on body temperature. An imbalance caused by an agonist or an antagonist of one of these molecular thermoregulators results in either transient hypothermia (lower body temperature) or transient hyperthermia (higher body temperature). Thermoregulatory effects of TRPM8 agonists and TRPV1 antagonists are similar resulting in a higher body temperature while TRPM8 antagonists and TRPV1 agonists cause a decrease in body temperature albeit to different extent.

A number of thermoTRP channels act as thermosensors in endothermic organisms and play a role in thermoregulation^17–21^. For example, both TRPM8 and TRPV1 have a level of basal activation (tone) that can balance thermoregulation^9,10,22^. Basal TRPV1 activation contributes to a suppressive tone on body temperature and further activation of TRPV1 by chemical agonists like capsaicin and resiniferatoxin (RTX) cause a decrease in body temperature (towards hypothermia). Basal TRPM8 activation contributes to an enhancing tone on body temperature and further activation of TRPM8 by cold or chemical agonists cause an increase in body temperature (towards hyperthermia). Conversely, TRPV1 antagonists cause an increase in body temperature^9,10,23^, whereas TRPM8 antagonists cause a reduction in body temperature (Fig. 3)^8–10,22^. Among the carriers of 10166942[C], reduced TRPM8 expression might functionally act as partial antagonism and keep a suppressive tone on body temperature.

**Figure 4:**
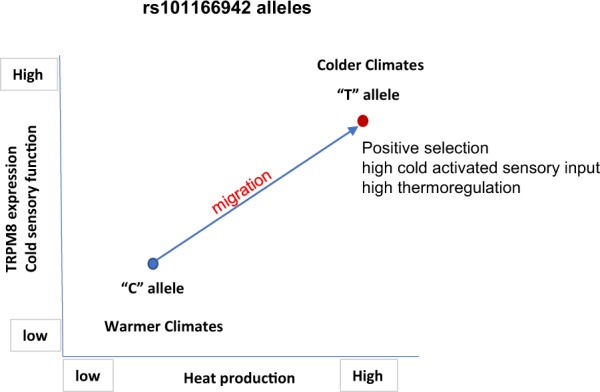
Working hypothesis - Evolutionary positive selection for “T” allele for rs10166942 is described in the context of TRPM8 expression, cold sensory function and the consequent thermoregulatory heat production. The ancestral “C” allele found among populations that inhabited warmer climates correlates with reduced TRPM8 function and attenuated cold sensory function. TRPM8 expression is higher among the carriers of “T” allele among inhabitants of colder climates and functionally might have provided higher cold activated sensory input that translates into higher thermoregulatory heat production resulted in the positive selection of this allele along the latitudinal cline as an adaptation to colder environments.

A recent evolutionary genetics study of the rs10166942 alleles found that the “C” allele is ancestral and infers a strong positive selection of the “T” allele along a latitudinal cline^24^, however, strong signals of association was not observed in an East Asian ancestry^25^. In fact, linkage analysis showed that the SNPs looked at in all these three studies (rs7577262, rs17862920, rs10166942) are linked (Supplementary Fig. 3). Here we showed that the ancestral “C” allele correlates with reduced TRPM8 expression and carriers of ancestral “C” allele have reduced cold and cold pain sensation. Intriguingly, the ancestral “C” allele is highest in frequency in populations in countries along the equatorial line where the highest atmospheric temperatures persist, thus the ancestral “C” allele may provide an evolutionary advantage to populations in these regions^24^. This advantage could manifest as a more suppressive tone on body temperature due to reduced cold sensor (TRPM8) expression that results in less heat production in hotter environments thus improved homeostatic management of body temperature (Fig. 4). In contrast, the positively selected “T” allele is in highest frequency in populations that inhabit colder climates^24^. The carriers of the “T” allele express higher levels of TRPM8 compared to the ancestral “C” allele, which may enable this population to maintain homeostatic thermoregulation with enhanced tone on body temperature in which more heat is produced that might have improved survival in colder climates (Fig. 4).

### Future directions

One limitation of this study is that the expression levels of TRPM8 in DRGs and QST/CPT studies were not conducted in the same cohort. TRPM8 expression was studied in DRGs from organ donors whereas QST/CPT was studied in a healthy volunteer cohort. However, individuals expected to have decreased TRPM8 expression in the healthy cohort Phase 0 study showed attenuated cold sensation and cold pain, thus revealing a clear functional consequence of reduced TRPM8 expression.

A second limitation of our study is that allelic expression imbalance has not been studied in migraine relevant trigeminal ganglia, but studied in DRG, which are more relevant to cold and cold pain sensation. Since DRGs were used as surrogates for trigeminal ganglia in this study, confirmation of these findings in human trigeminal ganglia is warranted.

A third limitation of our study is that while migraine is 3 times more prevalent in women, the QST and CPT in this study were performed in a predominantly male population. Avoiding estrus cycle effects was the reason for choosing to conduct the study in males; however, investigating the extent to which these results can be validated in females will be an important avenue for future investigation.

Another interesting finding was that the positive selection of rs10166942[T], which correlated with increased TRPM8 expression relative to rs10166942[C], suggests that an increased sensitivity to cold temperatures that results in more heat production in cold temperatures might have been a trait under selection in non-Africans that might have been important for survival in colder climates encountered north. A larger study with participants from Caucasian and African ancestry would avoid any ascertainment bias, and determining allelic expression imbalance and QST/CPT effects should further solidify the evolutionary interpretation.

## Methods

### TRPM8 allelic expression imbalance studies

#### Selection of marker single-nucleotide polymorphisms (mSNP)

Tissue specimens were collected within 4 hours postmortem and flash frozen in liquid nitrogen. To assess allele-specific mRNA expression, candidate mSNPs were identified using the database of SNPs (Database of Single Nucleotide Polymorphisms (dbSNP). Bethesda (MD): National Center for Biotechnology Information, National Library of Medicine (dbSNP Build ID: 141); available at: http://www.ncbi.nlm.nih.gov/SNP/), University of California Santa Cruz Genome Browser^26^ and Array Studio (OmicSoft Corporation, Research Triangle Park, North Carolina, USA). Selection criteria to determine the suitability of an mSNP were as follows: the mSNP must be located in the coding region of *TRPM8*; the mSNP must be within 30 kb of the non-coding reduced migraine risk SNP rs10166942 because downstream linkage analysis (phasing) techniques have certain limitations (e.g., using low quantities of genomic DNA [gDNA], long-range [LR] polymerase chain reaction [PCR] analysis and cloning of large DNA fragments); the mSNP must have high heterozygosity in the CEU population; and the mSNP must be amenable to PCR amplification, so that the mSNP-containing amplicon would fall within a single exon. mSNPs meeting these criteria are shown in Supplementary Table 1a.

#### Isolation of gDNA and total RNA

To extract gDNA, frozen DRG tissue (~15 mg) was suspended in DNA lysis buffer containing proteinase K at 55 °C overnight, and then purified using the Gentra Puregene Core Kit A and treated with RNase A according to the manufacturer’s instructions (Qiagen Inc., Valencia, California, USA). gDNA samples were stored in TE buffer (10 mM Tris-HCl pH 7.5, 1 mM EDTA) at −80 °C.

To isolate total RNA, frozen DRG tissue (~20 mg) was homogenized using a RNeasy Mini Kit (Qiagen) buffer and a rotor-stator homogenizer. Total RNA was then purified according to the manufacturer’s instructions. In brief, following resuspension in 50 µl of RNase-free water, contaminating gDNA was removed by incubating with 2 U of RNase-free DNase (Qiagen) at 37 °C for 60 minutes. The RNA was then purified using a Qiagen RNeasy Mini Kit and stored at −80 °C. gDNA and total RNA were quantified using a NanoDrop™ UV- Vis spectrophotometer (Thermo Fisher Scientific Inc., Waltham, Massachusetts, USA).

#### Genotyping

Genotyping of gDNA samples for SNPs rs11562975, rs13004520 and rs28901637 was performed using pyrosequencing on a PyroMark Q24 instrument (Qiagen). A single genotyping assay was used to test heterozygosity for these SNPs because of their position on the same 77-bp amplicon. The genomic region was amplified using the following primers: forward primer 5′-CCTCAATATCCCCTTCCC-3′, a biotinylated reverse primer 5′-GCAGCAACACTTGAAGCACTT-3′ and a sequencing primer 5′-ATCCCCTTCCCCTTCAT-3′. The template sequence used to generate a reagent-dispensing order was 5′-(C/G)AGATCC(A/T)CT(C/G)T(A/G)TATCCTGGACAACAACCACACACATTTG-3′.

For the pyrosequencing reactions, 15 ng of gDNA was mixed with forward and reverse primers and Phusion High-Fidelity PCR Master Mix (Thermo Fisher Scientific), and was amplified using the following thermocycling program: 1 cycle of 95 °C for 3 minutes; 35 cycles of 95 °C for 15 seconds, 62 °C for 15 seconds and 72 °C for 20 seconds; and followed by a final cycle of 72 °C for 5 minutes. The PCR products were then run on the pyrosequencer using the pre-programmed standard protocol, assay-specific sequencing primer and the supplied PyroMark Q24 template sequence to generate a reagent-dispensing order. The percentage of base calls at each position was identified using the pyrosequencing trace files in the PyroMark Q24 software v2.0.7, and genotyping calls were made based on the ratio of the expected alleles. Heterozygous calls were made if both alleles were present at 25% or greater ratios.

Genotyping of SNPs rs10166942 and rs17862920 was performed using allele-specific TaqMan^®^ qPCR assays on the Applied Biosystems 7900HT Fast Real-Time PCR System (Applied Biosystems Inc, Foster City, California, USA). The assays were purchased from Applied Biosystems (Catalog# 4351379; Assay IDs C__30341627_10 and C__2641482_10, respectively). A total of 15 ng of gDNA from each sample were loaded into the recommended qPCR reaction master mix and amplified for 40 cycles using the following standard qPCR thermocycling program: 1 cycle of 95 °C for 2 minutes; 40 cycles of 95 °C for 30 seconds and 60 °C for 1 minute. The data were collected after each cycle. A common threshold was set for all samples and the Cq values were recorded.

The ratio of alleles was calculated using the equation (2^(40-CqSNP))/((2^(40-CqREF)) + (2^(40-CqSNP)) with the CqSNP value corresponding to the Cq value of the minor SNP allele, and the CqREF value corresponding to the Cq value of the reference allele giving a value between 0 (reference allele) and 2 (minor allele). Heterozygous calls were made if the value calculated was between 0.5 and 1.5. Genotypes of samples used in this study are shown in Supplementary Table 1b.

#### Complementary DNA (cDNA) synthesis

Total RNA (1 µg) was treated with DNase I using the TURBO™ DNase Kit (Thermo Fisher Scientific). The RNA was diluted to 43 µl with nuclease-free water. A total of 5 µl of 10 × DNase I buffer and 2 µl of DNase I enzyme were added, and the samples were incubated at 37 °C for 1 hour. For the reaction inhibitor, 6 µl were added and mixed thoroughly at room temperature. DNase-treated RNA was purified using an RNeasy MinElute Cleanup Kit (Qiagen) and then eluted in 20 µl of nuclease-free water.

RNA was reverse transcribed using the SuperScript II Reverse Transcriptase kit (Invitrogen, Carlsbad, California, USA) following the manufacturer’s protocol. In brief, 2 µl of oligo d(T) and dNTP mix were added to each RNA sample, heated to 65 °C for 5 minutes in a thermocycler and then placed on ice for 2 minutes. For each reaction, 8 µl of 5× first-strand buffer, 4 µl of 0.1 M DTT and 2 µl of RNAseOUT™ were added. Of the reaction mixture, 10 µl were removed and set aside as a negative template control (RT−). The remaining reaction mixture (RT+) was placed in a pre-heated thermocycler at 42 °C for 2 minutes. A total of 2 µl of SuperScript II Reverse Transcriptase were added to each reaction directly and the samples were maintained at 42 °C for 50 minutes. The temperature was increased to 70 °C for 15 minutes; the samples were then removed and placed on ice. Of nuclease-free water, 45 µl and 15 µl were added to the RT + and RT− samples, respectively.

#### PCR amplification of gDNA and cDNA segments containing reduced migraine risk SNPs and mSNPs

The regions of gDNA containing the reduced migraine risk SNPs and mSNPs were amplified using PCR. Approximately 5–20 ng of gDNA isolated from the DRG samples were amplified using Phusion High-Fidelity PCR Master Mix (Thermo Fisher Scientific) following the manufacturer’s instructions. To reduce any potential for imbalance differences owing to PCR amplification bias, the total number of amplifications was limited to a maximum of 12 cycles. The oligonucleotide primers and PCR amplicon size are shown in Supplementary Table 1a. The regions of cDNA containing the mSNPs were amplified using the same oligonucleotide primers and PCR reaction conditions that were used for amplification of the gDNA regions.

#### Allelic imbalance sequencing

gDNA and cDNA amplicons were end-repaired using the End-It™ DNA End-Repair Kit (Epicentre Biotechnologies (an Illumina company), Madison, Wisconsin, USA) following the manufacturer’s instructions. Barcoded adapters from the TruSeq DNA-Seq Kit (Illumina, Inc., San Diego, California, USA) were ligated to the ends of the gDNA and cDNA amplicons following the manufacturer’s instructions. Barcoded amplicons were further amplified using limited-cycle PCR (7 cycles), and purified using Agencourt AMPure XP beads (Beckman Coulter, Inc., Brea, California, USA) following the manufacturer’s instructions. Barcoded DNAs, along with a 20% PhiX loading control, were sequenced using an Illumina MiSeq^®^ System as described in the Illumina MiSeq cluster generation and sequencing kits. The minimum number of reads required to assess allelic imbalance was no less than 10,000 reads per barcoded DNA.

#### Computational analysis

Sequencing data were mapped to the human genome (version B37.3) and further analyzed for SNP annotation and frequency using the Array Studio software package (OmicSoft).

Digital counts for each nucleotide variant were matched to the barcoded amplicon and mapped back to the gDNA and cDNA samples and individual DRG samples from which they were originally derived. Ratios of allelic imbalance were calculated as the number of reads for the minor allele divided by the number of reads for the common allele.

#### LR PCR and SNP phasing

rs10166942(C) and rs17862920(T) alleles with reduced migraine risk were phased with the mSNP alleles on the maternal and paternal chromosomes using LR PCR. Approximately 150 ng of gDNA from each donor were mixed with 0.2 µM oligonucleotide primers (5′-CACAGCCCCCACCCTTCCCTACTTAC-3′ and 5′-TGGCCGTATTTATCATTGCCTTTAGCATG-3′), GoTaq^®^ Long PCR Master Mix (Promega Corporation, Madison, Wisconsin, USA) and 0.5 M betaine. Thermocycling conditions for PCR were as follows: preheat at 65 °C for 5 minutes; followed by 95 °C for 2 minutes; then 18–26 cycles of 93 °C for 30 seconds and 65 °C for 29 minutes. A final extension was carried out at 72 °C for 15 minutes. The LR PCR products were subcloned into a phagemid using the GC Cloning and Amplification Kit (Lucigen Corporation, Middleton, Wisconsin, USA) following the manufacturer’s instructions. Alleles were assigned to chromosomes by performing Sanger sequencing at both ends of the LR PCR–cloned fragments using SP6 and T7 primers.

#### Source of human DRGs

All human dorsal root ganglia that were used for this study were obtained by legal consent from organ donors or next of kin in the US. Policies for donor screening and consent are the ones established by the United Network for Organ Sharing. Organizations supplying human tissues to AnaBios follow the standards and procedures established by the US Centers for Disease Control and are inspected biannually by the Department of Health and Human Services. Tissue distribution is governed by internal IRB procedures and compliance with HIPAA regulations regarding patient privacy. All organ donor transfers to AnaBios (https://anabios.com/contact/). are fully traceable and periodically reviewed by US Federal authorities. DRGs were acquired from fifteen (eight males, seven females) human adult organ donors (19–59 years of age) during the removal of the vital organs for transplantation. No information about the medication use or disease diagnoses were available.

### Study to evaluate the cardiovascular reactivity and cold pain tolerance (CPTo) of healthy volunteers with or without specific SNPs in the *TRPM8* gene

#### Study participants

Inclusion criteria were as follows: healthy males and females of non-childbearing potential (i.e., 1-year postmenopausal or surgically sterile), between the ages of 18 and 55 years, inclusive; body mass index between 18.0 and 32.0 kg/m^2^, inclusive, at screening; physical and neurological examinations, clinical laboratory values and electrocardiograms (ECG) that were clinically acceptable to the investigator and Amgen physician; screening and baseline blood pressures for all participants were 90–130 mm Hg for their systolic reading and 50–85 mm Hg for their diastolic reading; screening and baseline heart rate were 50–100 bpm; screening and baseline oral temperatures were 36.1–37.8 °C; respiratory rate at screening and baseline were 12–18 respirations/min; participants exhibited a mean increase in systolic and diastolic blood pressures of at least 10% above their baseline values in response to each cold pressor test (CPT) session; following completion of the screening CPT assessments, participant’s blood pressure returned to the normal range (90–130 mm Hg for their systolic reading and 50–85 mm Hg for their diastolic reading) within 5 minutes; participant understood study procedures and requirements, and provided written informed consent approved by an Independent Ethics Committee/Institutional Review Board (Quorum review, https://www.quorumreview.com/).

Exclusion criteria were as follows: history or evidence of a clinically significant disorder, condition or disease that, in the opinion of the investigator and Amgen physician, significantly impaired pain perception (e.g., history of stroke, history of neuropathy) or interfered with evaluation, procedures or study completion; prior or current history of peripheral neuropathy, paresthesias or dysesthesias; a history of hypertension, hypotension, symptomatic or asymptomatic coronary artery disease, percutaneous transluminal coronary angioplasty, coronary artery bypass surgery, peripheral vascular disease and any cerebrovascular event, including but not limited to cerebrovascular accident or transient ischemic attack; prior or current history of pulmonary or metabolic diseases (e.g., diabetes), or primary or secondary Raynaud’s disease or any other condition related to severe cold intolerance; participants exhibiting systolic blood pressure above 180 mm Hg or diastolic blood pressure above 110 mm Hg during the CPT at screening or baseline; CPTo of less than 120 seconds following the CPT during screening; participants unable to refrain from ingesting solid food for at least 2 hours before the start of the quantitative sensory testing (QST) and/or CPT (between study assessments on days 1 and 2, participants can have water, fruit juice, vegetable juice and light snacks [e.g., fruit, crackers]); participants unable to refrain from ingesting caffeinated, decaffeinated and/or alcoholic beverages during the 24 hours preceding testing; women who are pregnant; participants unable to refrain from exercise during the 24 hours prior to testing; one or both parents diagnosed with hypertension; prior or current history of psychiatric illness, such as anxiety, depression or schizophrenia; evidence of any current illness (e.g., common cold, viral syndrome, flu-like symptoms); evidence of any disturbance of the autonomic nervous system, including significant orthostatic hypotension and/or heart rate changes at screening exam (orthostatic hypotension defined as a systolic blood pressure decrease of at least 20 mm Hg or a diastolic blood pressure decrease of at least 10 mm Hg within 3 minutes of standing compared with blood pressure from the sitting or supine position); evidence of frostbite or bruising, cut, sore or fracture of the hand to be immersed; prior or current history of syncope or seizures; history of chronic or frequent clinically significant pain; current use of any prescription or non-prescription medication, including but not limited to opioids, benzodiazepines or other anxiolytics, tricyclic antidepressants or other antidepressants, anticonvulsants or botanical or herbal remedies; use of any investigational drug (small molecule) or device within 60 days prior to study enrollment; use of any investigational or therapeutic biologic (e.g., antibody) within <6 months prior to study enrollment; current or prior history of tobacco use or use of any other nicotine-containing product; active substance abuse within 12 months prior to study enrollment; positive for human immunodeficiency virus antibodies, hepatitis B surface antigen, hepatitis B core antibody or hepatitis C antibodies at screening; or individual previously had entered this study. Individuals provided written informed consent before participating in any study-specific activities or procedures.

#### Study design

This was a phase 0, parallel-group, method validation study conducted at a single center in the United States between May 20, 2013 (first participant enrolled) and June 16, 2013 (last participant completed study). The study planned to enroll approximately 40 participants.

The study comprised a 28-day screening period (day –28 to day –1), followed by a two- day assessment period. Each participant who entered the screening period received a unique identification number via manual assignment. During the screening period, SNP genotyping was performed after an initial screening CPT assessment had been completed.

Participants who met the screening criteria were enrolled and separated into two groups: carriers (+SNP) included participants with either SNP (rs10166942 or rs17862920) or both SNPs, and non-carriers (−SNP) included participants without either SNP. In Caucasian populations, T is considered the common allele at rs10166942 and C is the reduced risk allele. Conversely, C is considered the common allele at rs17862920, whereas T is the reduced risk allele^5^. During the two-day assessment period, QST and CPT were performed three times per day. The total planned study duration was 30 days.

#### Study procedures

All study tests and procedures were conducted at a room temperature maintained at 20 ± 2 °C. Participants refrained from ingesting solid food for at least 2 hours prior to screening and study procedures on days 1 and 2. Participants avoided alcohol, caffeinated and decaffeinated beverages during the 24 hours prior to assessments. Tobacco and nicotine use were not permitted throughout the screening period and for the duration of the study. Participants also avoided exercise during the 24 hours prior to assessments. Between study assessments on days 1 and 2, participants were allowed water, juice and light snacks.

Baseline vital signs were measured at screening and prior to both QST and CPT. Clinical laboratory assessments, ECGs and physical examinations were performed at screening.

Concomitant medications, procedure-related events and safety reporting were recorded throughout the study.

#### Study Approvals

Written informed consent was received from participants prior to inclusion in the study.

For the studies of allelic imbalance, all human dorsal root ganglia (DRG) specimens were collected under Institutional Review Board approval with appropriate informed consent. In all cases, materials obtained were surplus to standard clinical practice. Patient identity and protected health information/identifying information were redacted from tissues and clinical data. Donors were between the ages of 19 and 37 years. The study protocol and any amendments for the Phase 0 study to evaluate responsiveness of carriers of rs10166942[C] to cold challenge were approved by an Independent Ethics Committee/Institutional Review Board (Quorum review IRB, https://www.quorumreview.com/). The study was conducted in accordance with the International Conference on Harmonization Tripartite Guideline for Good Clinical Practice and applicable regulations and guidelines.

#### DNA genotyping

Reference DNA samples representing the three independent genotypes for each SNP (rs10166942 and rs17862920) were purchased from the Coriell Institute for Medical Research (Camden, New Jersey, USA). These samples served as blinded controls during assay validation and subsequently as process controls during clinical testing.

Clinical samples in the form of EDTA-preserved whole blood were collected at screening and stored frozen at −70 °C until shipped on dry ice for DNA extraction and genotyping (QuintilesIMS, Inc., Marietta, Georgia, USA). gDNA was extracted from 200 µl EDTA- preserved whole blood using the QIAcube system (Qiagen). DNA with an A260/280 ratio in the range of 1.7–2.0 was accepted as pure. DNA was genotyped for *TRPM8* SNPs rs10166942 and rs17862920 using TaqMan^®^ SNP genotyping assay ID C__30341627_10 and C__2641482_10 on the ViiA™ 7 Real-Time PCR system (Applied Biosystems). gDNA (10–15 ng) was amplified by TaqMan^®^ Genotyping Master Mix using the following thermocycling conditions: 1 cycle of 95 °C for 10 minutes; followed by 40 cycles of 95 °C for 15 seconds and 60 °C for 90 seconds. Data were analyzed by allele discrimination using the TaqMan^®^ software.

#### QST

The QST protocol of the German Research Network on Neuropathic Pain has been validated in 180 healthy volunteers and used to assess the somatosensory function in patients with neuropathic pain^27,28^.

QST was performed by placing a Peltier thermode (contact area of 5.0 × 2.5 cm^2^) against the skin (forearm) of the non-dominant arm. Thermal stimuli were delivered through the Peltier thermode using a computerized system^28^. The thermode was set at a baseline temperature of 32 °C (defined as thermal neutrality) and was cooled at a rate of 1 °C/s until a temperature of 0 °C was reached. This temperature was maintained for the remainder of the test (maximum duration 120 seconds). Participants pushed a button when their cold pain threshold (CPTh) and CPTo levels were reached. The times at which the participant experienced CPTh and CPTo were recorded, along with the intensity of the pain using the Likert scale.

QST was administered just prior to the CPT and performed on the contralateral upper limb in order to minimize desensitization. A 5-minute minimum recovery period between the end of QST and the start of the CPT ensured that the participant’s vital signs were in the normal range.

#### CPT

The CPT has been widely used to measure cardiovascular reactivity to environmental stress^29–31^. CPT stimulus involves both cold and pain elements and triggers an α-adrenergic vasoconstriction with an increased total peripheral resistance (5). The CPT was performed by immersing the participant’s dominant hand into an ice bath (1–5 °C) for up to 6 minutes, while blood pressure and heart rate were measured.

CPT was performed once during screening. Blood pressure and heart rate were measured every 60 seconds during the CPT and again 5 minutes after the participant removed their hand from the ice bath. After immersing their hand into the ice bath, participants were asked to indicate when the sensation first became painful (CPTh) by saying “painful,” and state when they were no longer able or willing to tolerate the pain (CPTo) by saying “stop.”

On assessment days 1 and 2, CPT was performed three times per day within 40 minutes of QST administration and with 2 hours between each set of assessments. Blood pressure and heart rate were measured every 60 seconds during the CPT and, when possible, immediately after the participant reached CPTo. Blood pressure and heart rate were also measured 5 minutes after the participant removed their hand from the ice bath in the first and second CPT assessments, and 10 minutes after the third CPT assessment. The time at which the participant experienced CPTh and CPTo was recorded, along with the intensity of the pain using the Likert scale.

An oral temperature was taken prior to starting QST and the CPT, immediately after the participant reached CPTo for both tests and 5 minutes after the completion of both tests.

After the final CPT of each day, every participant had a 10-minute minimum recovery period.

#### Study endpoints

The primary endpoint was the change in systolic and diastolic blood pressures in response to CPT. Secondary endpoints were CPTh and CPTo, as measured by the Likert scale (0–10) for the CPT and QST.

#### Statistical analyses

We planned to enroll approximately 40 participants in this study. This sample size was based on practical considerations; however, sufficient power existed (80%) to detect a difference between the 20 participants with either SNP (rs10166942 or rs17862920) or both SNPs, and the 20 participants without either SNP when the difference in systolic blood pressure between the two groups is 8.45 mm Hg following CPT (assuming a standard deviation of 9.5 mm Hg).

All participants with at least one post-baseline CPT or QST measure were included in the primary analysis set. For the analysis of the primary endpoint, change from baseline in blood pressure following CPT was analyzed using a repeated measures analysis of covariance.

Independent variables included SNP type, time (from CPT initiation) and the SNP type-by- time interaction. SNP type comprised the following three levels: homozygous for the risk allele; heterozygous with a risk allele and a protective allele; or homozygous for the protective allele. The baseline measures were used as a covariate and subject was used as a random effect. For each SNP combination, SNP type-by-time baseline-adjusted least squares mean with 95% confidence intervals and *P* values for the null hypothesis of no difference are presented. All repeated measures analyzed used the mixed-effects model repeated measures method to account for missing data.

For the analysis of the secondary endpoints, time to event analyses for both CPTh and CPTo in response to CPT and QST challenge were carried out using Kaplan–Meier survival and Cox regression models. Within-subject repeated measures of each event were first averaged prior to the analyses. For QST challenge, the maximal duration extended to 152 seconds and these events were right-censored. For CPT challenge, the maximal duration extended to 300 seconds and these events were right-censored. Time to event curves were generated for each of the three SNP types, and hazard ratios with corresponding *P* value were estimated by fitting a Cox regression model for the null hypothesis of no difference.

Temperature measurements for CPTh and CPTo in response to QST challenge were analyzed using a non-parametric Kruskal–Wallis test to compare mean ranks of CPTh or CPTo temperatures between SNP groups. In the event that the Kruskal–Wallis omnibus *P* value achieved significance, subsequent comparisons between SNP groups were made using the Dunn–Sidak method for multiple test corrections. Likert scores for both CPTh and CPTo in response to CPT and QST challenge were analyzed similar to temperature measurements. These analyses were performed in those participants that had either SNP or both SNPs (carriers) and those participants that had neither SNP (non-carriers), as well as separately for the SNP loci rs10166942 and rs17862920.

## Supplementary information

Supplementary Information
