# Supplementary material for: Reduced TRPM8 expression underpins reduced migraine risk and attenuated cold pain sensation in humans
Source: Sci Rep. 2019 Dec 23;9:19655. doi: 10.1038/s41598-019-56295-0 (PMC6927963; doi:10.1038/s41598-019-56295-0)
Supplement: Supplementary file 1 — Supplementary Information [file 41598_2019_56295_MOESM1_ESM.docx]

Supplementary information

**Reduced TRPM8 expression underpins reduced migraine risk and attenuated cold pain sensation in humans**

Narender R Gavva^1,^ *, Robert Sandrock^1^, Gregory E Arnold^1^, Michael Davis^1^, Edwin Lamas^1^, Chris Lindvay^1^, Chi-Ming Li^1^, Brian Smith^1^, Miroslav Backonja^2^, Kristin Gabriel^1^ & Gabriel Vargas^1^

1 Amgen Inc., Thousand Oaks, California, USA

2 Worldwide Clinical Trials, Morrisville, North Carolina, USA.

**SUPPLEMENTARY MATERIALS**

Supplementary Figure 1 Changes in systolic (a) and diastolic (b) blood pressures for the different *rs*10166942 genotype groups (T/T, T/C, C/C) during the cold pressor test.


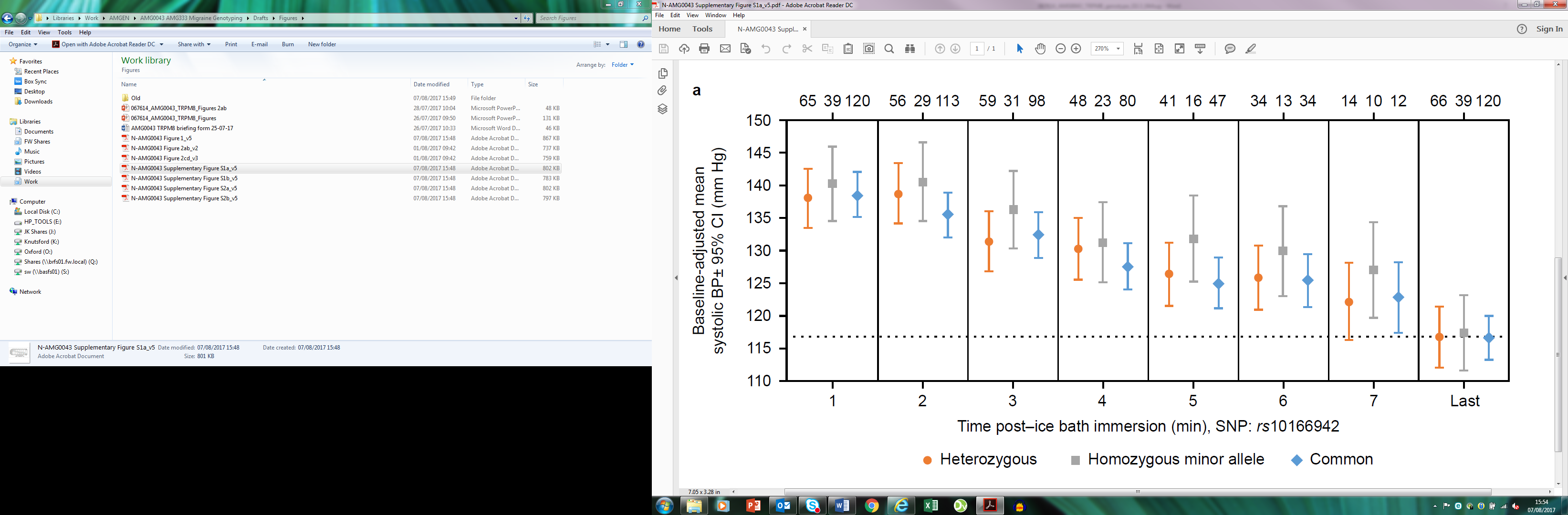


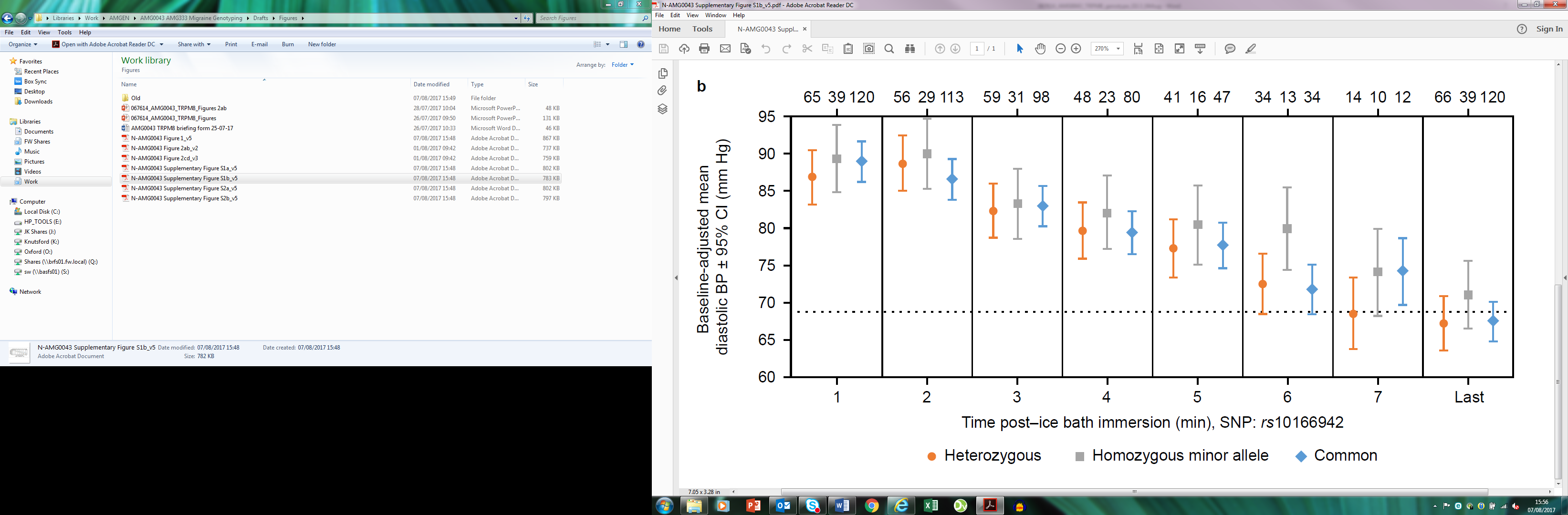


Baseline-adjusted mean blood pressure (BP) levels and their 95% confidence intervals (CI) are plotted by time post-immersion into an ice bath. The number of observations per single-nucleotide polymorphism (SNP) group (common, T/T; heterozygote, T/C; minor allele homozygote, C/C) is indicated at the top of the plots. The dotted line designates the mean baseline BP. A mixed-effects linear least squares model was used to fit the data with time and genotype as nominal factors, baseline as a covariate, a genotype*time interaction term and subject as a random factor.

Supplementary Figure 2 Changes in systolic (a) or diastolic (b) blood pressures for the different rs17862920 genotype groups (C/C, C/T, T/T) during the cold pressor test.


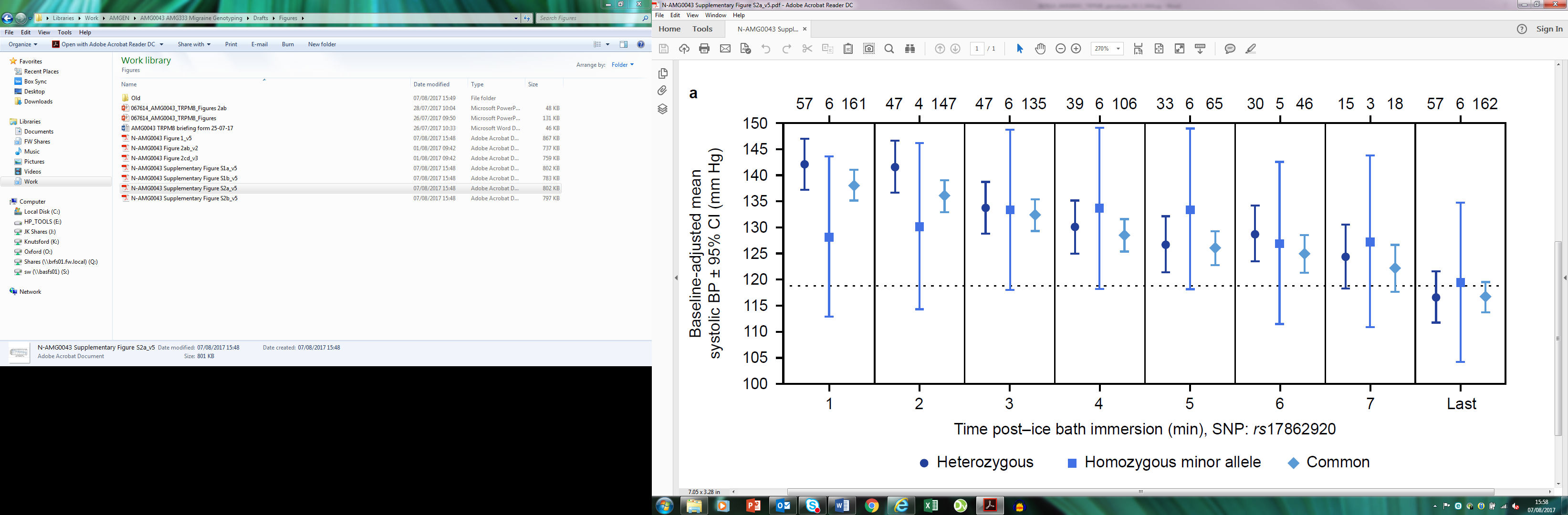


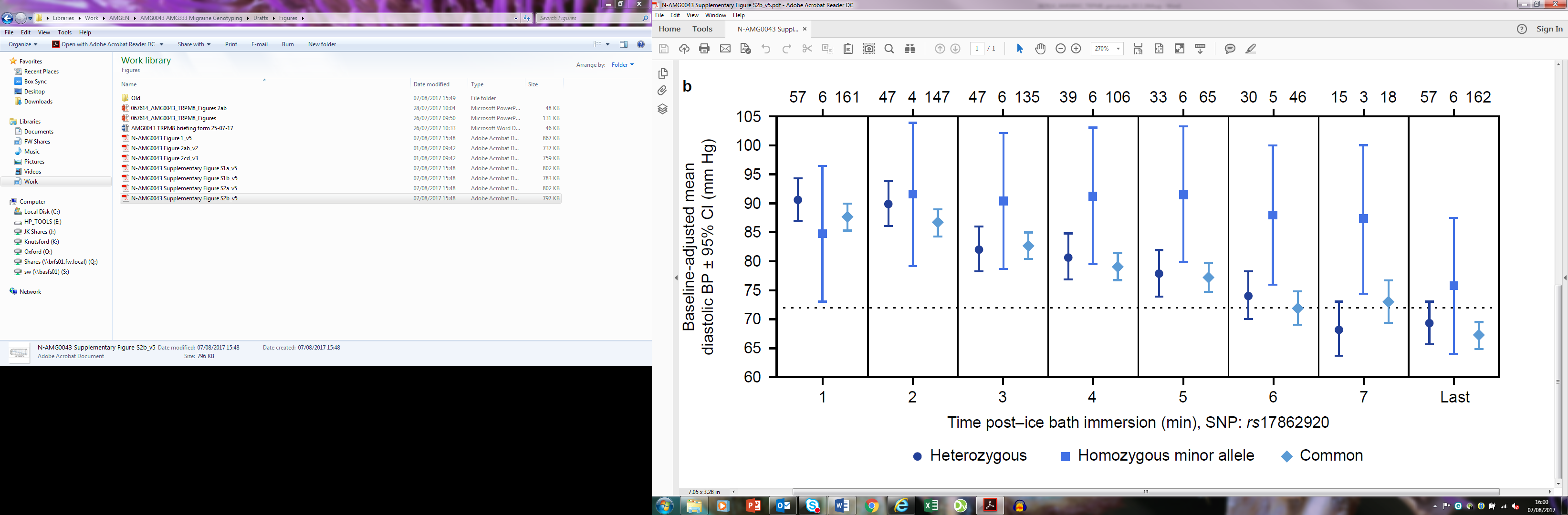


Baseline-adjusted mean blood pressure (BP) levels and their 95% confidence intervals (CI) are plotted by time post-immersion into an ice bath. The number of observations per single-nucleotide polymorphism (SNP) group (common, C/C; heterozygote, C/T; minor allele homozygote, T/T) is indicated at the top of the plots. The dotted line designates the mean baseline BP. A mixed-effects linear least squares model was used to fit the data with time and genotype as nominal factors, baseline as a covariate, a genotype*time interaction term and subject as a random factor.

**Supplementary Figure 3**: Linkage analysis for SNPs studied here and other studies are shown.

**Supplementary Table 1.** Description of reduced migraine risk–associated SNP alleles and mSNP alleles, allelic frequency, primers used to amplify genomic and cDNA regions, and length of PCR product.

| dbSNP ID: alleles | Het^b^ | Forward primer (5' to 3') | Reverse primer (5' to 3') | Length of PCR product (bp) |
| --- | --- | --- | --- | --- |
| rs10166942: T>C^a^ | 0.31 | CTTTATGACCTGTCTGAGCC | GGAAGGATAGGGTTGTAGTG | 139 |
| rs17862920: C>T | 0.20 | AATAGTACCACGGCCAAC | GAGACCCAATCTTAAACATTAAGC | 163 |
| rs11562975: G>C | 0.18 | GCCCAGTACCTTATGGATGA | GCTCAGAGATATACTTCTCTAGCTG | 151 |
| rs13004520: G>C | 0.12 | GCCCAGTACCTTATGGATGA | GCTCAGAGATATACTTCTCTAGCTG | 151 |
| rs28901637: A>T | 0.02 | GCCCAGTACCTTATGGATGA | GCTCAGAGATATACTTCTCTAGCTG | 151 |

^a^Allele with higher population frequency. ^b^Frequency of heterozygosity (het) in the CEU population. PCR, polymerase chain reaction; SNP, single-nucleotide polymorphism.

**Supplementary Table 2.** Genotypes of six DRG samples used in this study.

| Sample ID | rs10166942 | rs17862920 | rs11562975 | rs28901637 | rs13004520 |
| --- | --- | --- | --- | --- | --- |
| 379630 | 1 | 0 | 1 | 0 | 0 |
| 385164 | 1 | 0 | 1 | 0 | 0 |
| 390387 | 1 | 0 | 1 | 0 | 0 |
| 390388 | 1 | 0 | 0 | 1 | 0 |
| 397628 | 1 | 1 | 0 | 0 | 1 |
| 397629 | 1 | 0 | 1 | 0 | 0 |

Value of “1” denotes heterozygosity; value of “0” denotes homozygosity for the common allele. No samples were homozygous for the minor allele.

**Supplementary Table 3.** Phasing of alleles rs10166942 and rs17862920 associated with reduced migraine risk and marker alleles showing allelic expression imbalance

| DRG sample | Chromatid | rs10166942 | rs17862920 | mSNP rs11562975 | mSNP rs28901637 | mSNP rs13004520 | Number of clones sequenced^a^ |
| --- | --- | --- | --- | --- | --- | --- | --- |
| 385164 | 1 | **C**^b^ | C | **C**^c^ | A | G | 6 |
|  | 2 | T | C | G | A | G | 8 |
| 390387 | 1 | **C** | C | **C** | A | G | 16 |
|  | 2 | T | C | G | A | G | 17 |
| 390388 | 1 | **C** | C | G | **T** | G | 24 |
|  | 2 | T | C | G | A | G | 17 |
| 397628 | 1 | **C** | **T**^d^ | G | A | **C** | 9 |
|  | 2 | T | C | G | A | G | 8 |
| 397629 | 1 | **C** | C | **C** | A | G | 21 |
|  | 2 | T | C | G | A | G | 16 |

^a^Number of phagemid clones sequenced that show a particular linkage pattern. ^b^rs10166942 allele (C) associated with reduced migraine risk is shown in green. ^c^mSNP alleles associated with reduced allelic expression are shown in red. ^d^rs17862920 allele (T) associated with reduced migraine risk is shown in blue. DRG, dorsal root ganglia; mSNP, marker single-nucleotide polymorphism.

**Supplementary Table 4.** Screen summary of male volunteer genotypes for SNP rs10166942 and rs17862920

|  | | | Screen status | | | | | | | | |
| --- | --- | --- | --- | --- | --- | --- | --- | --- | --- | --- | --- |
|  |  |  | Pass | | | |  | Fail | | | |
| SNP | Genotype | SNP status | Count (*n*=70) | Presence (%) | Group | Presence (%) |  | Count  (*n*=95) | Presence (%) | Group | Presence (%) |
| rs10166942 | C/C | +SNP | 7 | 10 | A | 41 |  | 7 | 7 | A | 40 |
|  | C/T | +SNP | 22 | 31 |  |  |  | 31 | 33 |  |  |
|  | T/T | –SNP | 41 | 59 | B | 59 |  | 57 | 60 | B | 60 |
| rs17862920 | T/T | +SNP | 1 | 1 | A | 26 |  | 0 | 0 | A | 24 |
|  | T/C | +SNP | 17 | 24 |  |  |  | 23 | 24 |  |  |
|  | C/C | –SNP | 52 | 74 | B | 74 |  | 72 | 76 | B | 76 |

SNP, single-nucleotide polymorphism.

**Supplementary Table 5.** Analyses of time to temperature of threshold for SNP groups (rs10166942) during the CPT

|  | Carriers  +SNP  *N*=18 | Non-carriers  –SNP  *N*=20 |
| --- | --- | --- |
| Threshold (CPT) |  |  |
| LS geometric mean (seconds) | 18.03 | 15.78 |
| Ratio between SNP groups (95% CI) | 1.14 (0.61–1.68) | ― |
| *P* value^a^ | 0.5918 | ― |

The carrier group includes volunteers with either rs10166942(C) or both rs10166942(C) and rs17862920(T). ^a^*P* value is associated with +SNP group versus –SNP group.
Time to pain intolerance could not be adequately estimated as 35.4% (*n*/*N*=93/263) of observations were censored because subjects did not reach intolerance. CI, confidence interval; CPT, cold pressor test; LS, least squares; SNP, single-nucleotide polymorphism.

Supplementary Table 6. Comparison of CPT measurements between genotypes for the rs10166942 and rs17862920 loci

| rs10166942 | SNP groups | | | | | |  | Comparisons between SNP groups | | | | | |
| --- | --- | --- | --- | --- | --- | --- | --- | --- | --- | --- | --- | --- | --- |
|  | T/T | | T/C | | C/C | | Difference tested | T/T–T/C | | T/T–C/C | | T/C–C/C | |
|  | Median | *n* (obs) | Median | *n* (obs) | Median | *n* (obs) |  | Value  (95% CI) | *P* value | Value  (95% CI) | *P* value | Value  (95% CI) | *P* value |
| CPT time to threshold (seconds) | 12 | 20 (119) | 15 | 11 (66) | 43 | 7 (39) | Hazard ratio | 1.23  (0.58–2.64) | 0.59 | 0.41  (0.16–1.04) | 0.06 | 0.34  (0.11–1.03) | 0.06 |
| CPT time to tolerance (seconds) | 207 | 20 (120) | 300^a^ | 11 (66) | 209 | 7 (39) | Hazard ratio | 0.53  (0.19–1.47) | 0.23 | 0.93  (0.34–2.56) | 0.88 | 1.65  (0.48–5.72) | 0.43 |
| Likert score threshold | 3.0 | 20 (120) | 3.0 | 11 (66) | 2.0 | 7 (39) | Mean ranks^b^ | ― |  | ― |  | ― |  |
| Likert score tolerance | 7.5 | 20 (119) | 7.7 | 11 (66) | 6.0 | 7 (39) | Mean ranks^b^ | ― |  | ― |  | ― |  |

| rs17862920 | SNP groups | | | | | |  | Comparisons between SNP groups | | | | | |
| --- | --- | --- | --- | --- | --- | --- | --- | --- | --- | --- | --- | --- | --- |
|  | C/C | | C/T | | T/T | | Difference tested | C/C–C/T | | C/C–T/T | | C/T–T/T | |
|  | Median | *n* (obs) | Median | *n* (obs) | Median | *n* (obs) |  | Value  (95% CI) | *P* value | Value  (95% CI) | *P* value | Value  (95% CI) | *P* value |
| CPT time to threshold (seconds) | 15 | 27 (162) | 14 | 10 (57) | 43 | 1 (6) | Hazard ratio | 0.97  (0.45–2.08) | 0.93 | N/A |  | N/A |  |
| CPT time to tolerance (seconds) | 207 | 27 (162) | 209 | 10 (57) | 300^a^ | 1 (6) | Hazard ratio | 0.63  (0.24–1.68) | 0.59 | N/A |  | N/A |  |
| Likert score threshold | 2.00 | 27 (162) | 1.67 | 10 (57) | 1.33 | 1 (6) | Mean ranks^b^ | ― |  | ― |  | ― |  |
| Likert score tolerance | 4.92 | 27 (162) | 3.83 | 10 (57) | 1.83 | 1 (6) | Mean ranks^b^ | ― |  | ― |  | ― |  |

N/A indicates that no comparisons were made because *n*=1 in the T/T SNP group. ^a^Median was a censored event. ^b^Statistical significance was not achieved, therefore no comparisons between groups were made. CI, confidence interval; CPT, cold pressor test; SNP, single-nucleotide polymorphism.

**Supplementary Table 7.** Analysis of Likert Pain Scale data for SNP groups (rs10166942) during QST

|  | Carriers  +SNP  *N*=18 | Non-carriers  –SNP  *N*=20 |
| --- | --- | --- |
| Threshold (QST) |  |  |
| LS mean | 1.93 | 2.19 |
| Difference between SNP groups (95% CI) | –0.26 (–1.10, 0.57) | ― |
| *P* value^a^ | 0.54 | ― |
| Intolerance (QST) |  |  |
| LS mean | 3.59 | 4.78 |
| Difference between SNP groups (95% CI) | –1.19 (–2.59, 0.21) | ― |
| *P* value^a^ | 0.095 | ― |

The carrier group includes volunteers with either rs10166942(C) or both rs10166942(C) and rs17862920(T). ^a^*P* value is associated with +SNP group versus –SNP group. CI, confidence interval; LS, least squares; QST, quantitative sensory testing; SNP, single-nucleotide polymorphism.

**Supplementary Table 8.** Analysis of Likert Pain Scale data for SNP groups (rs10166942) during the CPT

|  | Carriers  +SNP  *N*=18 | Non-carriers  –SNP  *N*=20 |
| --- | --- | --- |
| Threshold (CPT) |  |  |
| LS mean | 3.13 | 3.20 |
| Difference between SNP groups (95% CI) | –0.07 (–1.02, 0.88) | ― |
| *P* value^a^ | 0.89 | ― |
| Intolerance (CPT) |  |  |
| LS mean | 6.40 | 6.90 |
| Difference between SNP groups (95% CI) | –0.50 (–1.85, 0.85) | ― |
| *P* value^a^ | 0.46 | ― |

The carrier group includes volunteers with either rs10166942(C) or both rs10166942(C) and rs17862920(T). ^a^*P* value is associated with +SNP group versus –SNP group. CI, confidence interval; CPT, cold pressor test; LS, least squares; SNP, single-nucleotide polymorphism.
